# A Novel ECG Score for Predicting Left Ventricular Systolic Dysfunction in Stable Angina: A Pilot Study

**DOI:** 10.3390/diagnostics16020237

**Published:** 2026-01-12

**Authors:** Nadir Emlek, Hüseyin Durak, Mustafa Çetin, Ali Gökhan Özyıldız, Elif Ergül, Ahmet Seyda Yılmaz, Hakan Duman

**Affiliations:** Department of Cardiology, Faculty of Medicine, Recep Tayyip Erdoğan University, 53020 Rize, Türkiye

**Keywords:** left ventricular systolic dysfunction, electrocardiography, fragmented QRS, R-wave peak time, stable angina pectoris, coronary artery disease

## Abstract

**Background:** Left ventricular systolic dysfunction (LVSD) is a major determinant of prognosis in patients with ischemic heart disease. Electrocardiography (ECG) is widely available, inexpensive, and may aid in identifying patients at risk. We hypothesized that a composite score derived from multiple established ECG markers could improve the detection of LVSD in patients with stable angina. **Methods:** In this single-center, cross-sectional study, 177 patients undergoing elective coronary angiography for stable angina were included. Patients were classified as LVSD-negative (*n* = 123) or LVSD-positive (*n* = 54) based on echocardiographic ejection fraction. ECG parameters, including fragmented QRS, pathologic Q waves, R-wave peak time, QRS duration, and frontal QRS–T angle, were assessed. Independent predictors of LVSD were identified using multivariate logistic regression. A composite ECG score was constructed by assigning one point to each abnormal parameter. Model robustness was evaluated using bootstrap resampling (1000 iterations) and 10-fold cross-validation. **Results:** Multivariable analysis identified prior stent implantation, fragmented QRS, pathological Q waves, R-wave peak time, frontal QRS–T angle (log-transformed), and QRS duration as independent predictors of LVSD. ROC analysis demonstrated good discriminatory performance for R-wave peak time (AUC 0.804), QRS duration (AUC 0.649), and frontal QRS–T angle (AUC 0.825) measurements. The composite ECG score showed a stepwise association with LVSD: a score of ≥2 yielded high sensitivity (88%) and negative predictive value (97%), whereas a score of ≥3 provided high specificity (100%) and positive predictive value (100%). Bootstrap resampling and cross-validation confirmed model stability and strong discriminatory performance (mean AUC, 0.964; accuracy, 0.91). **Conclusions:** A simple composite ECG score integrating multiple established ECG markers is associated with the robust detection of LVSD in patients with stable angina. Although not a substitute for echocardiography, this score may support early risk stratification and help identify patients who warrant further imaging evaluations. External validation in larger and more diverse populations is required before routine clinical implementation of this model.

## 1. Introduction

Although the prognosis of stable angina is generally more favorable than that of acute coronary syndromes, the annual incidence of non-fatal myocardial infarction and mortality remains approximately 2–3% [1]. Therefore, identifying higher-risk patients within this population remains clinically important, as early recognition of adverse prognostic features may influence monitoring strategies and therapeutic decision-making.

Left ventricular systolic dysfunction (LVSD) is a major determinant of morbidity, functional capacity, and long-term prognosis in patients with ischemic heart disease [2,3]. Importantly, the detection of LVSD has therapeutic implications, as timely initiation of evidence-based medical therapy—and, in selected patients, revascularization—can improve outcomes even in the absence of overt symptoms [4]. Transthoracic echocardiography is the reference standard for the diagnosis of LVSD; however, despite its widespread use, routine echocardiographic assessment in all patients with stable angina may not always be feasible in certain clinical settings or resource-limited environments [5,6].

In routine clinical practice, the 12-lead surface electrocardiogram (ECG) remains the most accessible initial diagnostic tool because of its low cost, widespread availability, and ease of interpretation [7]. Numerous ECG abnormalities, including pathologic Q waves, fragmented QRS complexes, prolonged QRS duration, delayed R-wave peak time, and widened frontal QRS–T angle, have been individually associated with myocardial injury, ventricular remodeling, and LVSD. However, the diagnostic performance of any single ECG parameter for identifying LVSD is limited, and prior studies have largely evaluated these markers in isolation [7].

The novelty of the present study lies in the structured integration of multiple established ECG markers into a single composite scoring system rather than identifying new individual ECG abnormalities. This approach is intended to reflect the ECG phenotyping of ischemic myocardial disease by combining complementary depolarization and repolarization abnormalities. To date, a comprehensive ECG-based score integrating these parameters has not been systematically evaluated for the detection of LVSD in patients with stable angina who have undergone coronary angiography.

Accordingly, this study aimed to develop and assess an ECG-derived composite score and evaluate its diagnostic performance for identifying LVSD in this specific clinical setting. Any potential implications for clinical triage or risk stratification should be considered exploratory and hypothesis-generating, pending validation in independent cohort studies.

## 2. Materials and Methods

This single-center, cross-sectional, observational study was conducted in the Department of Cardiology at Recep Tayyip Erdoğan University Hospital between September 2024 and July 2025. A total of 129 male and 48 female consecutive patients who underwent elective coronary angiography with a preliminary diagnosis of stable angina pectoris (SAP) were included in the study.

All participants provided written informed consent prior to their enrollment. The study protocol was reviewed and approved by the institutional ethics committee in accordance with the principles of the Declaration of Helsinki (Approval Date: 15 August 2024, Approval No: 2024-196).

### 2.1. Patient Selection and Exclusion Criteria

Only patients aged 18–80 years who underwent elective coronary angiography with a preliminary diagnosis of stable angina pectoris (SAP) were included. Stable angina was defined as exertional chest pain without acute coronary syndrome (ACS). Patients with acute coronary syndrome, malignancy, chemotherapy or radiotherapy exposure, chronic kidney disease (estimated glomerular filtration rate [eGFR] < 30 mL/1.73 m^2^/min), hepatic failure, prior coronary artery bypass grafting or valve surgery, atrial fibrillation, hypertrophic cardiomyopathy, cardiac amyloidosis, congenital heart disease, a history of antiarrhythmic drug use (Vaughan–Williams class I or III), electrolyte imbalance, pacemaker presence, or pericardial effusion were excluded.

### 2.2. Clinical and Demographic Data Collection

A case report form containing information on demographic and clinical details, including conventional cardiovascular risk factors and current treatments, was completed during the initial visit. All patients underwent detailed medical history assessment, complete blood count, and serum biochemical testing. Data regarding patients’ medical histories, physical examinations, 12-lead ECGs, echocardiographic and coronary angiographic findings, concomitant systemic diseases, and medications were collected. Demographic variables, such as age, sex, diabetes mellitus (DM), hypertension (HT), dyslipidemia, history of coronary artery disease (CAD), prior percutaneous coronary intervention, and smoking status, were evaluated. Current guideline definitions were used for the diagnosis of DM, dyslipidemia, and HT [8,9,10]. Height and weight measurements were obtained, and the body mass index (BMI) was calculated using the formula: body weight (kg)/height squared (m^2^).

### 2.3. Laboratory Measurements

Hemogram and biochemical analyses were performed on peripheral venous blood samples obtained after fasting for at least 8 h using standard collection tubes. Baseline fasting glucose and creatinine levels were measured using chemiluminescence on an automated analyzer. Hemoglobin, white blood cell, and platelet counts were determined using samples collected in tubes anticoagulated with EDTA and analyzed using an automated hematology analyzer (Beckman Coulter, Brea, CS, USA).

### 2.4. Transthoracic Echocardiography

All patients underwent a comprehensive two-dimensional transthoracic echocardiographic evaluation performed by an experienced clinical cardiologist using a Philips Epiq 7 system equipped with a 1–5 MHz X5-1 transducer (Philips, Andover, MA, USA). Echocardiographic examinations were performed and interpreted by a single cardiologist who was blinded to the patients’ ECG findings and clinical symptoms. Images were obtained with the subjects in the left lateral decubitus position during normal respiration using parasternal long- and short-axis views and apical four- and two-chamber views with simultaneous ECG monitoring. All recordings included at least three cardiac cycles and were digitally stored for offline analyses. Standard two-dimensional and color Doppler flow images were obtained according to the American Society of Echocardiography guidelines. Ejection fraction was measured using the Simpson’s method [11]. LVSD was defined as a left ventricular ejection fraction (LVEF) of <50%.

### 2.5. Coronary Angiography

All selective coronary angiographies were performed under local anesthesia via the right femoral artery using manual contrast injection according to the Judkins technique. Angiographic images were obtained from multiple projections to ensure optimal visualization of the coronary artery. For each patient, the left anterior descending and left circumflex coronary arteries were imaged in at least four projections and the right coronary artery in at least two projections. Coronary angiograms were digitally recorded for quantitative analyses. Obstructive CAD was defined as ≥50% luminal stenosis in at least one major epicardial vessel on the index angiogram; prior revascularization was reported separately as stent history.

### 2.6. Electrocardiogram

A standard 12-lead surface ECG (150 Hz filter, 25 mm/s, 10 mm/mV; Schiller Cardiovit AT-10, Baar, Switzerland) was recorded for all patients before coronary angiography. The ECGs were scanned using a high-resolution scanner and magnified fivefold for detailed assessment. All ECG measurements were performed by a single experienced cardiologist who was blinded to the clinical, echocardiographic, and angiographic data. Predefined measurement criteria were uniformly applied to reduce the intra-observer variability.

The QRS duration was measured manually using digital calipers, defined as the interval from the onset of the q or R wave to the end of the S wave (J point), and the longest value among V1–V6 was recorded. A prolonged QRS duration was defined as a QRS interval of ≥120 ms. Right and left bundle branch blocks (RBBB and LBBB, respectively) were identified according to the standard electrocardiographic criteria. The frontal plane QRS and T axes were obtained from automated ECG analysis and manually confirmed when necessary.

Frontal QRS–T angle was calculated as the absolute difference between the frontal QRS and T axes; values > 180° were adjusted to an acute angle by subtracting from 360°. A fragmented QRS was defined as the presence of additional notches or deflections within the QRS complex, indicating nonhomogeneous ventricular depolarization. Pathologic Q waves were evaluated in contiguous leads and defined as Q waves ≥ 40 ms in duration and ≥25% of the amplitude of the subsequent R wave.

The P-wave indices were measured in leads where the waveform was clearly visualized. The maximum and minimum P-wave durations were determined, and the P-wave dispersion was calculated as their difference. The R-wave peak time was measured as the interval from the onset of the QRS complex to the peak of the R-wave. The QT interval was measured from the beginning of the Q wave to the end of the T-wave. The corrected QT interval (QTc) was calculated using Bazett’s formula (QTc = QT/√RR). All ECGs were scanned using a standardized high-resolution protocol and analyzed at fixed magnification. Measurements were performed using digital calipers by a single experienced cardiologist blinded to all clinical and echocardiographic data. Predefined measurement criteria were uniformly applied to all ECGs to minimize intra-observer measurement variability.

### 2.7. Statistical Analysis

All statistical analyses were performed using the SPSS software (version 26, SPSS Inc., Chicago, IL, USA). Continuous variables are expressed as mean ± standard deviation for normally distributed data and as median with interquartile range (25th–75th percentiles) for non-normally distributed data. Categorical variables are presented as frequencies and percentages. Normality of continuous variables was assessed using visual inspection (histograms and probability plots) and analytical tests (Kolmogorov–Smirnov and Shapiro–Wilk tests).

Between-group comparisons were conducted using Student’s *t*-test for normally distributed continuous variables and the Mann–Whitney U test for non-normally distributed variables. Categorical variables were compared using the chi-square test or Fisher’s exact test, as appropriate.

Associations between clinical and ECG variables and LVSD were evaluated using univariate logistic regression. Prior to multivariable modeling, multicollinearity among candidate predictors was assessed using the variance inflation factor (VIF) and tolerance statistics; variables with evidence of collinearity were not entered simultaneously into the same model.

Variables that demonstrated statistical significance in the univariate analysis (*p* < 0.05) were entered into the multivariable logistic regression model. Given the total number of LVSD events (*n* = 54), the number of variables was deliberately limited to maintain an acceptable events-per-variable (EPV) ratio and reduce the risk of overfitting. C-reactive protein (CRP) and other non-normally distributed variables were logarithmically transformed prior to modeling, when appropriate.

Bootstrap and Cross-Validation: model robustness was assessed using bootstrap resampling with 1000 iterations. The model performance was further evaluated using 10-fold stratified cross-validation. The discriminatory ability was quantified using the area under the receiver operating characteristic curve (AUC), and the classification accuracy was calculated for each fold.

Diagnostic Performance of the ECG Score: the diagnostic performance of the composite ECG Score was evaluated using logistic regression-derived predicted probabilities. The pre-test probability was defined as the overall prevalence of LVSD in the study population. Calibration was assessed by comparing the observed and predicted event rates across score categories. The optimal cutoff values were determined using the Youden index. Likelihood ratios were calculated to estimate the post-test probabilities. Sensitivity, specificity, positive predictive value (PPV), and negative predictive value (NPV) were calculated for individual ECG parameters and the composite score.

Pairwise comparisons of ROC curves were performed to compare the discriminative ability of the composite ECG score with that of the individual electrocardiographic parameters. Differences in the AUC values were assessed using a non-parametric bootstrap approach with 2000 resamples. This method was chosen because of the potential separation and non-normal score distributions; therefore, DeLong’s test was not applied.

Intra-Observer Reliability: The intra-observer reliability of the ECG measurements was assessed using the intraclass correlation coefficient (ICC). An ICC ≥ 0.75 was considered indicative of good-to-excellent reliability.

## 3. Results

A total of 177 patients (129 men and 48 women; mean age, 57.7 ± 13 years) were included in the analysis. Based on echocardiographic findings, participants were categorized into the LVSD (−) (*n* = 123) and LVSD (+) (*n* = 54) groups (Table 1). Patients with LVSD were significantly older and had a higher cardiometabolic burden. Compared with the LVSD (−) group, those with LVSD (+) had higher fasting glucose, serum creatinine, white blood cell, and neutrophil counts, and CRP levels, while hemoglobin and albumin levels were lower. Hypertension, diabetes mellitus, dyslipidemia, prior stent implantation, established coronary artery disease, male sex, and use of β-blockers and oral antidiabetic/insulin therapy were more prevalent in the LVSD (+) group.

**Figure 1:**
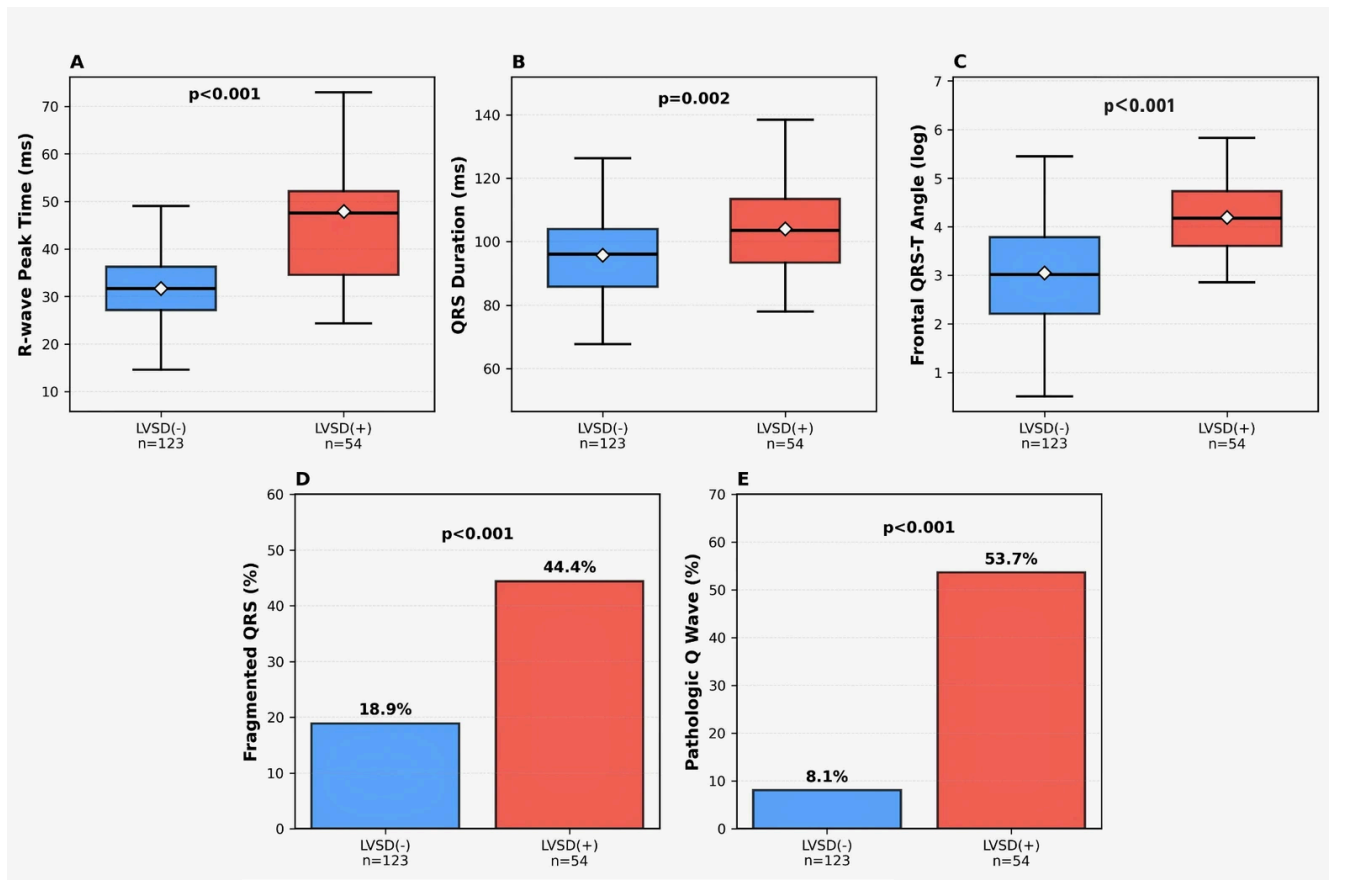
Comparison of ECG parameters in patients with and without LV systolic dysfunction. Box plots show (**A**) R-wave peak time, (**B**) QRS duration, and (**C**) frontal QRS-T angle. Bar charts display prevalence of (**D**) fragmented QRS and (**E**) pathologic Q waves. Boxes represent the interquartile range, the horizontal line represents the median, the symbol inside the box indicates the mean, whiskers indicate data dispersion. LVSD: Left ventricular systolic dysfunction.

The electrocardiographic characteristics differed significantly between the groups (Table 2). Patients with LVSD showed a higher prevalence of fragmented QRS complexes, pathological Q waves, and left bundle branch blocks. Depolarization and repolarization parameters were markedly altered in the LVSD (+) group, including a prolonged R-wave peak time, increased QRS duration, and greater deviation of the QRS and T axes, resulting in a significantly wider frontal QRS–T angle. These between-group differences are illustrated in Figure 1, and the key ECG parameters are summarized in Table 3.

Univariate logistic regression analysis identified multiple clinical and electrocardiographic variables associated with LVSD (Table 4). Non-normally distributed variables, including the frontal QRS–T and C-reactive protein (CRP) levels, were log transformed prior to analysis. Variables that demonstrated significant between-group differences were evaluated using univariate logistic regression analysis (Table 4). The remaining significant parameters were subsequently entered into a backward multivariable logistic regression model.

The final multivariable model identified prior stent implantation, fragmented QRS, pathological Q waves, R-wave peak time, frontal QRS–T angle (log), and QRS duration as independent predictors of LVSD. Given the total number of LVSD events (*n* = 54), the resulting events-per-variable ratio was 9, indicating an acceptable model complexity and a low risk of overfitting. Multicollinearity among the electrocardiographic predictors was assessed using variance inflation factors (VIF). After excluding the composite ECG score, all VIF values were <2, indicating no relevant multicollinearity among the individual ECG parameters. This supports the interpretation of the ECG score as a composite variable integrating complementary rather than redundant electrocardiographic information.

Internal validation using bootstrap resampling with 1000 iterations confirmed the stability of regression coefficients. In bootstrap analyses, the composite ECG score remained a strong independent predictor of LVSD (bootstrap-derived OR 17.6, 95% CI 7.75–49.20), whereas clinically established coronary artery disease also retained a robust association (Table 5). Age showed a weaker and non-significant association after resampling.

Model performance was further evaluated using 10-fold stratified cross-validation. The model demonstrated excellent discriminatory ability, with a mean AUC of 0.96 and a high classification accuracy across the folds (Table 6).

**Figure 2:**
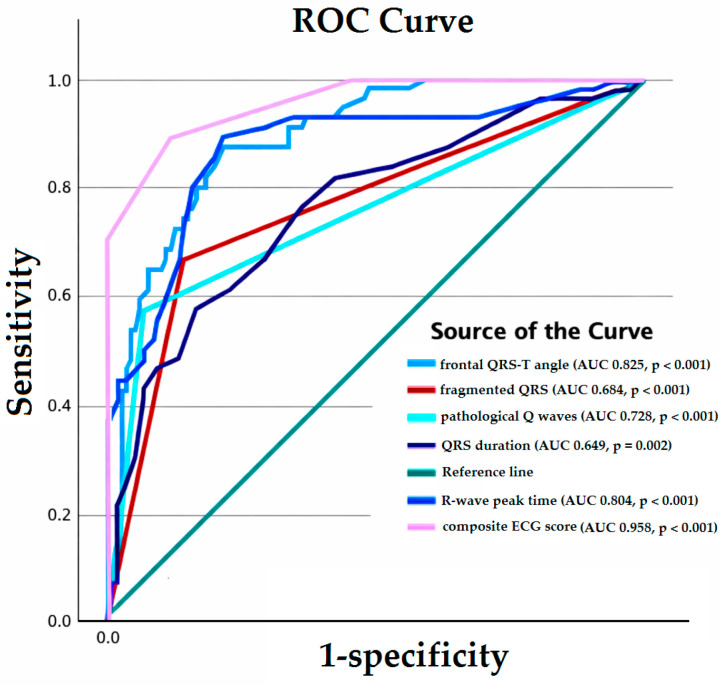
Receiver operating characteristic (ROC) curves demonstrating the diagnostic performance of individual ECG parameters and the composite ECG score for the detection of left ventricular systolic dysfunction (LVSD). ROC curves are shown for R-wave peak time, QRS duration, fragmented QRS, pathologic Q waves, frontal QRS–T angle (log-transformed), and the composite ECG score (0–5 points). The composite ECG score demonstrated superior discriminative ability compared with individual classical ECG markers, indicating incremental diagnostic value beyond single ECG abnormalities. Area under the curve (AUC) values are provided for each parameter.

ROC analyses were performed for the independent continuous electrocardiographic predictors of left ventricular systolic dysfunction (LVSD) (Figure 2). QRS duration, R-wave peak time, pathological Q waves, fragmented QRS, and frontal QRS–T angle demonstrated significant discriminatory ability for LVSD. The optimal cut-off values were determined using the Youden index and identified as QRS duration > 93 ms, R-wave peak time > 35.5 ms, and logarithmically transformed frontal QRS–T angle > 3.5.

The composite ECG score demonstrated superior discriminatory performance compared with classical ECG markers alone (pathologic Q waves, fragmented QRS, and bundle branch block), indicating an incremental diagnostic value (Table 7). The area under the ROC curve (AUC) values for individual ECG parameters were as follows: QRS duration (AUC 0.649, *p* = 0.002), R-wave peak time (AUC 0.804, *p* < 0.001), pathologic Q waves (AUC 0.728, *p* < 0.001), fragmented QRS (AUC 0.684, *p* < 0.001), and frontal QRS–T angle (AUC 0.825, *p* < 0.001) (Figure 2). Pairwise ROC comparisons demonstrated that the composite ECG score had significantly higher discriminatory performance than all individual electrocardiographic parameters, including R-wave peak time, frontal QRS–T angle (log), fragmented QRS, QRS duration, and pathologic Q waves. All differences in AUC (ΔAUC) favored the composite score and remained statistically significant based on the bootstrap-derived confidence intervals (Table 8).

**Figure 3:**
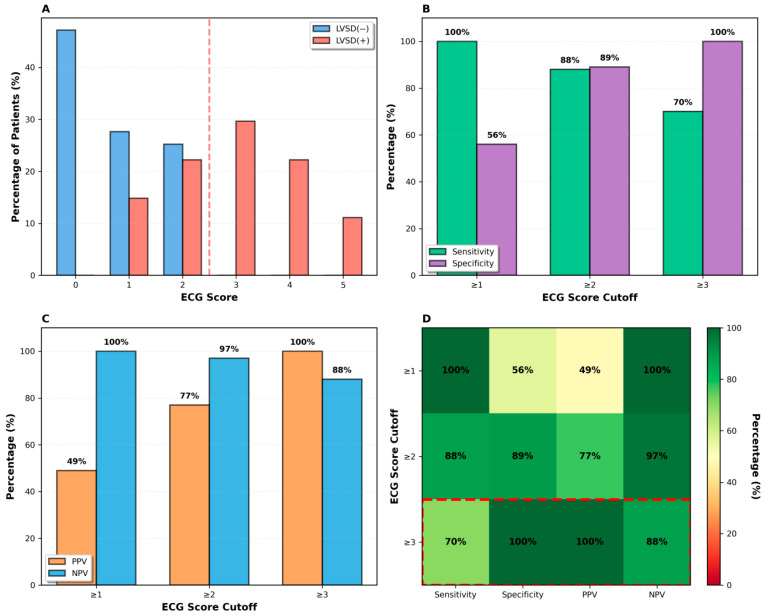
Diagnostic performance of the novel ECG score. (**A**) Score distribution by group with optimal cutoff (red dashed line). (**B**) Sensitivity and specificity at different cutoffs. (**C**) Positive and negative predictive values. (**D**) Comprehensive performance heatmap highlighting optimal cutoff (red box). The score ranges from 0 to 5 based on five ECG parameters. LVSD: Left ventricular systolic dysfunction; NPV: Negative predictive value; PPV: Positive predictive value.

Based on these findings, a composite ECG-based scoring system was constructed by assigning one point to each abnormal ECG parameter (Table 9). The composite score demonstrated a clear stepwise association with LVSD. In this cohort, a score ≥ 1 identified all LVSD cases, yielding a very high sensitivity and negative predictive value, although the specificity was limited. A score of ≥2 provided an optimal balance between sensitivity and specificity, with a sensitivity of 88%, specificity of 89%, positive predictive value of 77%, and negative predictive value of 97%. A score ≥ 3 was associated with very high specificity and positive predictive value, with reduced sensitivity (70%) and a negative predictive value of 83% (Figure 3).

Measurement Reliability; Intra-observer reliability of the ECG measurements was excellent, with an intraclass correlation coefficient (ICC) of 0.93, confirming the reproducibility of the ECG parameter assessment. Fagan Nomogram and Post-Test Probabilities; using the study population prevalence of LVSD (36%) as the pre-test probability, application of the composite ECG score allowed estimation of post-test probabilities. A positive test (Score ≥ 2) increased the likelihood of LVSD to approximately 81%, whereas a negative test reduced it to 5%, as illustrated by a Fagan nomogram

Overall, lower scores (0–2) were associated with high negative predictive values (88–100%), whereas higher scores (≥3) showed a strong positive predictive performance within the study population.

## 4. Discussion

In this study, fragmented QRS, pathologic Q waves, prolonged R-wave peak time, increased frontal QRS–T angle, and longer QRS duration were independently associated with LVSD in patients undergoing coronary angiography for stable angina. By integrating these ECG abnormalities into a composite ECG score, we demonstrated a clear stepwise relationship between increasing score values and the presence of LVSD in the study population. These findings indicate that combining multiple ECG parameters provides greater discriminatory power than evaluating individual ECG markers.

Several components of the proposed score represent well-established electrocardiographic manifestations of myocardial injury and LVSD. Pathologic Q waves reflect prior myocardial infarction [12], whereas fragmented QRS has been associated with myocardial fibrosis and scarring [13,14,15]. Prolonged QRS duration and abnormalities in depolarization–repolarization parameters, such as R-wave peak time and frontal QRS–T angle, likely reflect intraventricular conduction delay and electrical heterogeneity accompanying adverse ventricular remodeling [16,17]. The prevalence of obstructive CAD (67%) in our cohort is consistent with prior studies of patients with stable angina referred for invasive coronary angiography, reflecting a selected population with a high pre-test probability of ischemic heart disease. Within this context, the lower prevalence of angiographically obstructive CAD observed in the LVSD group should be interpreted cautiously, as this definition is based on the index angiogram and may not fully capture lifetime ischemic burden, including prior infarction or revascularized disease. Against this background, the strength of the present approach lies not in identifying novel individual ECG markers but in the structured integration of these complementary abnormalities into a single composite ECG phenotype. Equal weighting of each ECG parameter was deliberately chosen to preserve simplicity and clinical usability, particularly given the pilot nature of this study and the modest sample size. While regression-based weighting may improve discrimination in larger cohorts, an unweighted scoring approach reduces the risk of overfitting and facilitates transparent bedside implementation. Previous ECG-based approaches to LVSD prediction have largely focused on single parameters and complex modeling strategies [7]. In contrast, the present study proposes a simple, transparent, and clinically interpretable scoring system that combines multiple well-established ECG abnormalities, thereby leveraging their complementary diagnostic values.

Importantly, this ECG-based scoring system is not intended to replace established diagnostic modalities, such as transthoracic echocardiography or coronary angiography. Rather, it provides an integrated electrical phenotype that reflects the underlying myocardial damage, conduction delay, and repolarization abnormalities. As such, the score should be viewed primarily as a phenotyping and hypothesis-generating tool rather than a management-changing diagnostic instrument for routine clinical practice.

In the present cohort, a score ≥ 3 was associated with very high specificity for LVSD, suggesting its potential utility as a rule-in indicator in selected patients. However, the NPV is strongly influenced by disease prevalence, and the observed performance may not be generalizable to populations with different LVSD prevalence. Therefore, these findings should be interpreted within the context of the studied population and require confirmation in independent cohorts in future studies. Because both the ROC-derived cutoff values and the composite ECG score were developed and evaluated within the same cohort, the potential for overfitting cannot be excluded. Accordingly, the observed diagnostic performance should be interpreted with caution, and internal validation using resampling techniques such as bootstrapping, as well as external validation in independent cohorts, is required before it can be applied in a broader clinical setting. From a clinical perspective, different score thresholds may be interpreted pragmatically rather than prescriptively as follows: a score ≥ 2, which demonstrates a favorable balance between sensitivity and specificity, may be considered a reasonable trigger for echocardiographic evaluation in patients with stable angina. A score ≥ 3 represents a high likelihood (“rule-in”) zone, in which LVSD is highly probable within the studied population. Conversely, low scores should be interpreted cautiously and in conjunction with clinical pre-test probability; in patients with a high clinical suspicion of LVSD, a low score should not delay definitive imaging.

Overall, our results support the concept that composite ECG-based approaches may enhance the characterization of LVSD-related electrical remodeling beyond single ECG abnormalities. Future prospective multicenter studies are warranted to validate the score, assess its performance across different clinical settings, and clarify its potential role in clinical risk stratification.

The very high AUC observed for the composite score should be interpreted with caution, as it reflects internal discrimination in a single-center derivation cohort. Because the score and its cutoffs were derived and tested within the same dataset, some degree of optimistic performance is possible despite bootstrap resampling and cross-validation. External validation and recalibration in independent populations are therefore required before clinical implementation.

## 5. Limitations

This study had several limitations. First, it was conducted at a single center with a relatively small sample size, which may restrict the generalizability of our findings. In addition, the extensive exclusion criteria may limit the generalizability and potentially overestimate the diagnostic performance by studying a selected population with interpretable ECGs. The modest number of events may have led to model instability and optimistic estimates of discrimination.

Second, the scoring system was developed solely in patients with stable angina undergoing coronary angiography; therefore, its predictive performance may not be applicable to other clinical settings, such as non-ischemic cardiomyopathy or valvular heart disease, necessitating further validation in broader patient populations. Third, because all ECG and echocardiographic measurements were performed by a single experienced cardiologist, inter-observer variability could not be assessed. In addition, the analysis was based on digitally scanned and enlarged ECG recordings, a process that may introduce measurement variability despite a standardized evaluation. The cross-sectional design of the study also precludes the establishment of causal relationships between ECG parameters and the presence of LVSD. Moreover, LVSD was defined exclusively using LVEF obtained by the Simpson method, and other indices of systolic function, such as global longitudinal strain, were not evaluated. Although multivariate analysis was performed, residual confounding from comorbidities could not be entirely excluded. Finally, ROC-derived cutoff values and the resulting ECG-based scoring system were generated from the same cohort without internal or external validation, which may increase the risk of model overfitting. Accordingly, the proposed rule-in and rule-out thresholds were derived from a single cohort and require external validation before broader clinical application. In addition, the very high AUC may partly reflect optimistic model performance due to derivation and evaluation within the same cohort and the single-center case-mix. Furthermore, ECG findings were evaluated in a population in whom coronary angiography and echocardiographic imaging had already been performed; therefore, the present analysis did not assess the performance of the ECG score as a pre-imaging screening or triage tool. In addition, this study was not designed to evaluate whether the application of the ECG-based score influenced clinical decision-making, imaging strategies, or patient management. Larger prospective multicenter studies are required to confirm the clinical utility and external applicability of this scoring system.

## 6. Conclusions

In conclusion, our ECG-derived scoring system demonstrated excellent diagnostic performance for identifying LVSD in patients with ischemic CAD, suggesting a potential role in early risk stratification. Because ECG is widely available, inexpensive, and routinely used in clinical practice, incorporating multiple ECG markers into a structured score may assist in identifying patients who warrant further evaluation. While these findings contribute to the expanding interest in ECG-based diagnostic approaches, validation in larger and more diverse cohorts is needed before the score can be considered for broader clinical applications.

## Figures and Tables

**Table 1:** Baseline Clinical, Medication, and Laboratory Characteristics.

Variable	LVSD (−) (*n* = 123)	LVSD (+) (*n* = 54)	*p*
Sex (Male), *n* (%)	83 (67.5%)	46 (85.2%)	0.010
Age (years)	55.1 ± 13.2	63.9 ± 10.1	<0.001
Hypertension, *n* (%)	58 (47.2%)	35 (64.8%)	0.030
Dyslipidemia, *n* (%)	94 (76.4%)	48 (88.9%)	0.040
Diabetes Mellitus, *n* (%)	24 (19.5%)	22 (40.7%)	0.003
Stent History, *n* (%)	33 (26.8%)	30 (55.6%)	<0.001
* Obstructive CAD on index angiography (≥50% stenosis in ≥1 major epicardial vessel), *n* (%)	100 (81.3%)	19 (35.2%)	<0.001
BMI (kg/m^2^)	29.1 ± 4.7	28.9 ± 4.1	0.695
Smoking, *n* (%)	51 (41.3%)	24 (44.4%)	0.712
LVEF (%)	58.9 ± 3.7	40.01 ± 7.5	<0.001
Aspirin, *n* (%)	40 (32.5%)	34 (63%)	<0.001
P2Y12 Inhibitor, *n* (%)	3 (2.4%)	4 (7.4%)	0.128
ACE Inhibitor, *n* (%)	28 (22.8%)	17 (31.5%)	0.154
ARB, *n* (%)	24 (19.5%)	11 (20.4%)	0.524
Beta-blocker, *n* (%)	28 (22.8%)	25 (46.3%)	0.002
Calcium Channel Blocker, *n* (%)	11 (8.9%)	6 (11.1%)	0.420
Statin, *n* (%)	19 (15.4%)	11 (20.4%)	0.277
OAD/Insulin, *n* (%)	22 (17.9%)	21 (38.9%)	0.004
Glucose (mg/dL)	108.1 ± 33.3	135.1 ± 57.5	<0.001
Serum Creatinine (mg/dL)	0.89 ± 0.26	1.01 ± 0.22	0.009
Albumin (g/dL)	4.22 ± 0.27	3.9 ± 0.59	<0.001
WBC (×10^3^/µL)	8.01 ± 2.2	9.4 ± 2.6	<0.001
Neutrophils (×10^3^/µL)	5.8 ± 2.01	6.6 ± 2.5	<0.001
Lymphocytes (×10^3^/µL)	2.26 ± 0.75	2.04 ± 1.1	0.120
Hemoglobin (g/dL)	14.2 ± 1.8	13.5 ± 1.9	0.014
CRP (mg/L), median (IQR)	3.5 (2.5–5.3)	5.9 (3.5–9.5)	0.004
Ln-CRP	0.56 ± 0.28	0.76 ± 0.34	<0.001

Values are expressed as mean ± standard deviation or *n* (%), unless otherwise indicated. * This variable reflects angiographic findings at the index procedure and does not capture prior CAD treated by PCI, which is reported separately as ‘stent history’. CRP is presented as median (interquartile range). ACE, angiotensin-converting enzyme; ARB, angiotensin receptor blocker; BMI, body mass index; CAD, coronary artery disease; LVEF, left ventricular ejection fraction; OAD, oral antidiabetic drug; P2Y12, adenosine diphosphate P2Y12 receptor inhibitor; WBC, white blood cell count. *p* < 0.05 was considered statistically significant.

**Table 2:** Electrocardiographic Parameters.

Variable	LVSD (−) (*n* = 123)	LVSD (+) (*n* = 54)	*p*
Fragmented QRS, *n* (%)	23 (18.9%)	24 (44.4%)	<0.001
Prolonged QRS, *n* (%)	7 (5.7%)	9 (16.7%)	0.019
Wide QRS, *n* (%)	11 (8.9%)	15 (27.8%)	0.002
Pathologic Q Wave, *n* (%)	10 (8.1%)	29 (53.7%)	<0.001
RBBB, *n* (%)	8 (6.5%)	4 (7.4%)	0.826
LBBB, *n* (%)	3 (2.4%)	9 (16.7%)	0.001
QRS Axis (°)	21.1 ± 14.2	32.2 ± 23.5	<0.001
T Axis (°)	50 ± 37.7	99.8 ± 57.4	<0.001
Frontal QRS-T Angle, median (IQR)	21 (12–35)	108 (33.7–146)	<0.001
Frontal QRS-T Angle (log)	3.04 ± 1.1	4.3 ± 0.8	<0.001
QRS Duration (ms)	95 ± 13.5	102.7 ± 17.1	0.002
P-wave Duration Max (ms)	103.1 ± 6.6	104.4 ± 9.2	0.306
P-wave Duration Min (ms)	57.1 ± 5.1	58.6 ± 8.1	0.154
P-wave Dispersion (ms)	45.9 ± 4.06	46.1 ± 4.54	0.879
QT Interval (ms)	396 ± 34.3	387 ± 39.4	0.144
QTc Interval (ms)	416.6 ± 25.7	421.7 ± 30.6	0.263
R-wave Peak Time (ms)	32.3 ± 8.9	44.9 ± 12.8	<0.001

Values are expressed as mean ± standard deviation, median (interquartile range), or *n* (%), as appropriate. *p* < 0.05 was considered statistically significant. IQR, interquartile range; LBBB, left bundle branch block; QRS, QRS complex duration; QT, QT interval; QTc, corrected QT interval; RBBB, right bundle branch block.

**Table 3:** Key ECG Parameters Associated With LVSD.

Parameter	LVSD (−)	LVSD (+)	*p*-Value	Clinical Meaning
R-wave Peak Time (ms)	32.3 ± 8.9	44.9 ± 12.8	<0.001	Delayed activation
QRS Duration (ms)	95 ± 13.5	102.7 ± 17.1	0.002	Conduction delay
QRS–T Angle (log)	3.04 ± 1.1	4.3 ± 0.8	<0.001	Electrical desynchrony
Fragmented QRS (%)	18.9%	44.4%	<0.001	Myocardial scarring
Pathologic Q Wave (%)	8.1%	53.7%	<0.001	Prior MI

**Table 4:** Univariate and Multivariate Logistic Regression Analysis.

Variable	Univariate OR	95% CI	*p*	Multivariate OR	95% CI	*p*
* Sex (Male)	2.771	1.196–6.420	<0.001			
* Age (years)	1.061	1.030–1.093	<0.001			
* Hypertension	2.064	1.066–3.999	0.032			
Dyslipidemia	2.469	0.959–6.352	0.061			
* Diabetes Mellitus	2.836	1.405–5.725	0.004			
Stent History	3.409	1.747–6.654	<0.001			
* Coronary Artery Disease	17.27	5.11–58.343	<0.001	6.515	1.355–31.32	0.019
Aspirin	3.527	1.807–6.886	<0.001			
Beta-blocker	2.925	1.490–5.779	0.002			
OAD/Insulin	2.921	1.428–5.975	0.003			
Glucose	1.014	1.006–1.022	0.001			
* Serum Creatinine	4.949	1.399–17.513	0.013			
* Albumin	0.081	0.023–0.291	<0.001			
* WBC	1.270	1.105–1.459	0.001			
Neutrophils	1.352	1.161–1.574	<0.001			
* Hemoglobin	0.813	0.686–0.963	0.017			
Ln-CRP	9.189	2.806–30.095	<0.001			
* Fragmented QRS	5.380	2.664–10.865	<0.001	3.124	1.060–9.309	0.039
Fragmented QRS (with BBB)	3.228	1.340–7.776	0.009			
Prolonged QRS	3.314	1.164–9.433	0.025			
Wide QRS	3.916	1.659–9.246	0.002			
* Pathologic Q Wave	13.10	5.663–30.33	<0.001	6.5416	1.355–31.32	0.001
* LBBB	8.022	2.072–30.884	0.003			
* QRS Axis	1.035	1.016–1.054	<0.001			
* T Axis	1.022	1.014–1.031	<0.001			
* R-wave Peak Time	1.119	1.075–1.165	<0.001	1.139	1.060–9.309	<0.001
* Frontal QRS-T Angle (log)	3.572	2.363–5.398	<0.001	2.135	1.185–3.848	0.012
* QRS Duration	1.033	1.011–1.056	0.004	0.924	0.876–0.974	0.003

BBB: Bundle branch block; CI: Confidence interval; CRP: C-reactive protein; LBBB: Left bundle brunch block; OAD: Oral antidiabetic drug; OR: Odds ratio; QRS: QRS complex; WBC: White blood cell. Variables with marked with “*” entered into the multivariate logistic regression model (backward selection). Ln-CRP was used due to non-normal distribution of CRP values. *p* < 0.05 was considered statistically significant.

**Table 5:** Bootstrap-derived odds ratios for left ventricular systolic dysfunction.

Variable	OR (Bootstrap)	95% Bootstrap CI
Score	17.6	7.75–49.20
Age	1.04	0.98–1.11
Coronary Artery Disease	7.98	1.89–33.68

**Table 6:** Model performance based on 10-fold stratified cross-validation.

Metric	Mean	Standard Deviation
AUC	0.964	0.048
Accuracy	0.91	0.10

**Table 7:** Diagnostic performance of individual ECG parameters and the composite ECG score for LVSD.

Variable	Sensitivity (%)	Specificity (%)	Positive Predictive Value (%)	Negative Predictive Value (%)
QRS Duration > 93 ms	73	56	46	88
QRS Duration > 101 ms	52	80	68	92
R-wave Peak Time > 34.5 ms	83	63	58	95.6
R-wave Peak Time > 35.5 ms	77	74	66	96
R-wave Peak Time > 37.5 ms	66	79	72	96
Pathologic Q Wave	52	93	59	92
Fragmented QRS	55	82	67	85
Frontal QRS-T Angle (log) > 3.2	80	60	53	95
Frontal QRS-T Angle (log) > 3.5	75	74	63	96
Frontal QRS-T Angle (log) > 4.5	65	85	74	96
Score ≥ 1	100	56	49	100
Score ≥ 2	88	89	77	97
Score ≥ 3	70	100	100	88

Sensitivity, specificity, positive predictive value (PPV), and negative predictive value (NPV) were calculated for each ECG parameter using ROC-derived cut-off values. Frontal QRS–T angle refers to the log-transformed measurement used in regression analyses. Cut-off values were selected based on optimal Youden index unless otherwise stated. PPV and NPV are expressed as percentages.

**Table 8:** Pairwise bootstrap-based comparisons of AUC values between the composite ECG score and individual ECG parameters.

Comparison	ΔAUC	95% Bootstrap CI	*p*-Value
Score vs. QRS duration	0.20	0.13–0.27	<0.001
Score vs. Frontal QRS–T angle (log)	0.06	0.02–0.12	0.003
Score vs. Fragmented QRS	0.19	0.13–0.26	<0.001
Score vs. R-wave peak time	0.09	0.03–0.15	<0.001
Score vs. Pathologic Q wave	0.20	0.13–0.27	<0.001

**Table 9:** ECG-Based Scoring System (0–5 points).

ECG Parameter	Criterion	Score
R-wave peak time	>35.5 ms	+1
QRS duration	>101 ms	+1
Frontal QRS–T angle (log)	>3.5	+1
Fragmented QRS	Present	+1
Pathologic Q wave	Present	+1

## Data Availability

The datasets generated and/or analyzed during the current study are available from the corresponding author upon reasonable request but are not publicly available due to privacy and ethical restrictions.
